# Genome-Wide Characterization and Expression Profiling of the *GRAS* Gene Family in Salt and Alkali Stresses in *Miscanthus sinensis*

**DOI:** 10.3390/ijms232314521

**Published:** 2022-11-22

**Authors:** Xuhong Zhao, Yan Xu, Guo He, Kang He, Liang Xiao, Ruibo Hu, Shengjun Li

**Affiliations:** 1 CAS Key Laboratory of Biofuels, Shandong Provincial Key Laboratory of Energy Genetics, Qingdao Institute of Bioenergy and Bioprocess Technology, Chinese Academy of Sciences, Qingdao 266101, China; 2 Shandong Energy Institute, Qingdao 266101, China; 3 Qingdao New Energy Shandong Laboratory, Qingdao 266101, China; 4 College of Bioscience and Technology, Hunan Agricultural University, Changsha 410128, China

**Keywords:** soil salinization, *GRAS* gene family, *Miscanthus sinensis*, salt and alkali stress

## Abstract

The *GRAS* family genes encode plant-specific transcription factors that play important roles in a diverse range of developmental processes and abiotic stress responses. However, the information of *GRAS* gene family in the bioenergy crop Miscanthus has not been available. Here, we report the genome-wide identification of *GRAS* gene family in *Micanthus sinensis*. A total of 123 *MsGRAS* genes were identified, which were divided into ten subfamilies based on the phylogenetic analysis. The co-linearity analysis revealed that 59 *MsGRAS* genes experienced segmental duplication, forming 35 paralogous pairs. The expression of six *MsGRAS* genes in responding to salt, alkali, and mixed salt-alkali stresses was analyzed by transcriptome and real-time quantitative PCR (RT-qPCR) assays. Furthermore, the role of *MsGRAS60* in salt and alkali stress response was characterized in transgenic Arabidopsis. The *MsGRAS60* overexpression lines exhibited hyposensitivity to abscisic acid (ABA) treatment and resulted in compromised tolerance to salt and alkali stresses, suggesting that *MsGRAS60* is a negative regulator of salt and alkali tolerance via an ABA-dependent signaling pathway. The salt and alkali stress-inducible *MsGRAS* genes identified serve as candidates for the improvement of abiotic stress tolerance in Miscanthus.

## 1. Introduction

Soil salinization is becoming a major global environmental concern, which substantially affects the productivity of major crops and causes the deterioration of the ecological system [1]. Global saline soil has reached 424 million hectares, covering 3.9% of the land area in 2021 [2]. Moreover, the area of saline soil keeps increasing at a rate of 1–2 million hectares per year, and it is predicted to further increase due to changing environmental factors such as global warming and climate change [3]. How to alleviate land salinization and improve crop productivity in saline soil has always been an important research topic. Although a lot of physical, chemical, and engineering practices have been implemented in the reclamation of saline soil, they are faced with various shortcomings, such as cost-ineffective, laborious, non-environment friendly, and reversible reclamation effects. As an attractive alternative, the biological measures are more economical and environment-friendly for the reclamation of saline soil [4,5]. One of the biological measures of saline soil reclamation is the improvement of plant salt stress tolerance through various breeding techniques (e.g., molecular breeding) [6]. However, identification and functional characterization of key genes regulating salt stress response is a prerequisite for the genetic improvement of salt stress tolerance through breeding.

Miscanthus is a perennial C_4_ grass deemed as an ideal bioenergy crop for cellulosic bioethanol production. Miscanthus has several outstanding characteristics, such as high photosynthesis efficiency, large biomass yield, superior stress tolerance, and wide adaptability to various environments, which warrant it being one of the most promising bioenergy crops in European countries and China [7]. Therefore, it has wide adaptability in a broad range of tropical and temperate regions and yields a huge amount of lignocellulosic biomass [8,9]. Cultivation of Miscanthus on marginal land (e.g., saline soil) that is not arable for agronomic crops will not only provide sufficient raw materials for the second-generation bioethanol production, but will also help in the reclamation of saline soils, reduction of CO_2_ emissions, and carbon sequestration and neutralization [10,11]. However, compared with the main crops, the genetic improvement of Miscanthus lags far behind because of its complex genetic background, and the unclarified molecular mechanisms underlying agronomic traits and abiotic stress tolerance [12]. Recently, the genomic sequences of three Miscanthus species (i.e., *Miscanthus sinensis*, *Miscanthus floridulus*, and *Miscanthus lutarioriparius*) have been released [13,14,15], which significantly facilitates the identification of candidate genes governing important agronomic traits and stress tolerance in Miscanthus. The availability of these genomic resources is expected to accelerate the application of molecular breeding approaches in this important bioenergy crop.

The *GRAS* family genes encode plant-specific transcription factors named after the first three discovered members, i.e., GAI (gibberellic acid-insensitive), RGA (repressor of GAI), and SCR (scarecrow) [16]. The C-terminus of GRAS proteins is highly conserved, while the N-terminus is relatively variable. The conserved C-terminal domain consists of LHRI (Leucine Heptad Repeat I), LHRII, VHIID, PFYRE, and SAW subdomains [16,17]. Based on the conserved domains, GRAS members are divided into eight subfamilies including SCL3, SHR, PAT1, LISCL, DELLA, SCR, LAS, and HAM [17]. *GRAS* genes play important roles in diverse physiological and developmental processes including gibberellin acid (GA) signaling, meristem initiation and maintenance, and phytochrome A signaling [18]. Recent evidence indicates that GRAS transcription factors are involved in regulating plant abiotic stress responses. For example, a subset of *GRAS* genes in model plant species, such as Arabidopsis (*Arabidopsis thaliana*), rice (*Oryza sativa*), and tomato (*Solanum lycopersicum*) are documented to be involved in drought and salt stress tolerances. For instance, the DELLA proteins play vital roles in the response to cold and salt stresses by negatively regulating plant GA signaling in Arabidopsis [19,20,21]. *AtSCL14* and *OsGRAS23*, members of the LISCL subfamily from Arabidopsis and rice, are both positive regulators of drought stress tolerance [22,23,24]. *SlGARS7* and *SlGRAS10* from the PAT1 subfamily, and *SlGRAS40* from the HAM subfamily, are involved in drought and salt stress responses in tomato. When ectopically expressed, the transgenic lines confer enhanced drought and salt stress tolerances in tomato [25,26,27]. Apart from these *GRAS* genes, an increasing number of *GRAS* genes from other plant species such as poplar (*Populus euphratica* Oliv), rapeseed (*Brassica napus*), and soybean (*Glycine max*) have been identified to be involved in tolerance against a variety of abiotic stresses. For example, the poplar *GRAS* gene *PeSCL7* from the SCL subfamily confers enhanced drought and salt stress tolerances when overexpressed in Arabidopsis [28]. Overexpression of *BrLAS* from rapeseed results in improved drought tolerance in Arabidopsis [29]. Likewise, overexpression of *GmGRAS37*, a member of the LISCL subfamily, leads to significantly improved salt stress tolerance in transgenic soybean [30].

Despite that *GRAS* genes play important roles in plant development and abiotic stress responses, genome-wide identification and systematic analysis of the *GARS* gene family in Miscanthus has not been performed to date. Here we report the genome-wide identification of *GRAS* gene family in Miscanthus. A comprehensive analysis of Miscanthus *GRAS* gene family including sequence phylogeny, chromosomal location, segmental duplication, gene and protein structure, and expression profiling in saline and alkaline stress response are presented. In addition, we functionally characterize *MsGRAS60* in response to salt and alkali stresses in transgenic Arabidopsis. Our results enrich the understanding of the roles of *GRAS* genes in plant abiotic stress responses. The salt and alkali stress-inducible *GRAS* genes identified provide valuable candidates for future functional characterization of their roles in abiotic stress tolerance, which is expected to contribute to the improvement of Miscanthus abiotic stress tolerance via genetic engineering.

## 2. Results

### 2.1. Genome-Wide Identification of GRAS Genes in M. sinensis

The GRAS protein sequences were identified by local BLAST search against the annotated genome of *M. sinensis* using the HMM profile of the GRAS domain. The presence of the GRAS domain was further confirmed with the conserved domain (CD) search at NCBI. Meanwhile, sequences encoding less than 100 amino acids in length were manually excluded. Ultimately, 123 *GRAS* genes were identified in *M. sinensis* genome. They were designated *MsGRAS1* to *MsGRAS123* based on the physical locations on chromosomes. Detailed information of *M. sinensis GRAS* genes and their closest Arabidopsis orthologs are provided in Table 1. Generally, there were more than two *MsGRAS* orthologs for each Arabidopsis *GRAS* gene.

### 2.2. Chromosomal Locations of MsGRAS Genes

**Figure 1:**
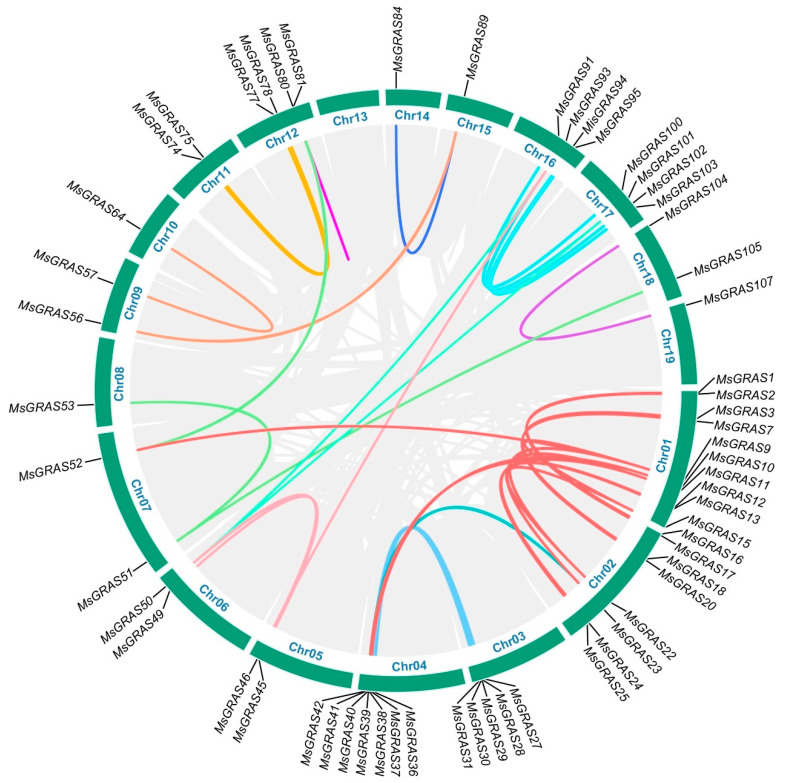
Segmental duplication relationships of *MsGRAS* genes. Grey lines indicate all synteny blocks in the *M.sinensis* genome. Lines in the same colors indicate duplicated GRAS gene pairs. The chromosomes are marked with Chr01 to Chr19 in blue for each chromosome.

Mapping of *MsGRAS* genes to chromosomes revealed that 108 of the 123 (87.8%) *MsGRAS* genes were located unevenly on 18 chromosomes, except for chromosome 13 (Figure 1 and Figure S1), while the rest were located on non-assembled scaffolds. The number of *MsGRAS* genes varied among different chromosomes. For example, there were 15 *MsGRAS* genes on chromosome 1, while only one gene was identified on chromosomes 8, 10, 14, 15, and 19, respectively. The distributions of *MsGRAS* genes on the chromosome were also not evenly arranged. For example, the *MsGRAS* genes on chromosomes 3 and 4 were concentrated in a small genomic area forming gene clusters. By contrast, the distributions of *MsGRAS* genes on chromosome 1 were relatively scattered.

To explore whether *MsGRAS* genes experienced duplication during the evolutionary process, we performed a co-linearity analysis based on the sequence homology of *MsGRAS* genes. The *MsGRAS* genes located in the same segmental duplication regions were shown in the Circos diagram (Figure 1). A total of 59 *MsGRAS* genes, accounting for 55% of the total genes with chromosome locations, were located in segmental duplication regions derived during the evolution of *M. sinensis*. The largest number of 16 *MsGRAS* genes derived from segmental duplications were found between chromosomes 1 and 2. These results indicated that the expansion of *MsGRAS* gene family is largely attributed to segmental duplication.

The segmental duplication event experienced by *MsGRAS* led to the formation of paralogous gene pairs. We identified a total of 35 paralogous pairs for *MsGRAS* genes (Table 2). The protein sequence identity between two members in the paralogous pairs ranged from 59.68% (*MsGRAS9*/*MsGRAS15*) to 98.44% (*MsGRAS29*/*MsGRAS39*). Substitution rates between non-synonymous (Ka) and synonymous (Ks) sites for each paralogous pair were lower than 1.0, suggesting that the paralogous pairs experienced purifying selection pressure during the evolution. The evolutionary divergence time for each paralogous pair was predicted. *MsGRAS12*/*MsGRAS25* and *MsGRAS45*/*MsGRAS49*, the latest duplicated paralogous gene pairs with protein identity of 98.09% and 98.25%, respectively, occurred approximately 0.67 million years ago (Mya). By contrast, *MsGRS51*/*MsGRAS105*, the earliest duplicated gene pair with a protein identity of 63.73%, diversified about 103.83 Mya.

### 2.3. Phylogenetic Relationships, Gene Structure, and Conserved Motifs of MsGRAS

**Figure 2:**
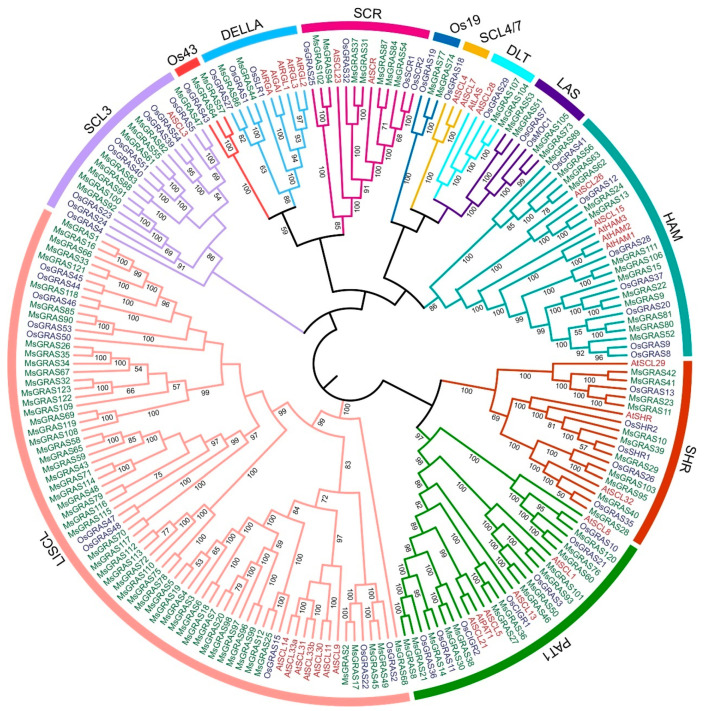
Phylogenetic relationships of GRAS proteins in *M. sinensis*, Arabidopsis, and rice. The un-rooted phylogenetic tree was generated by neighbor-joining (NJ) method with 1000 bootstrap replicates. Proteins were clustered into 12 clades and illustrated by specific colors belonging to the same subfamily. Bootstrap values larger than 50% were shown. GRAS members from *M. sinensis*, Arabidopsis, and rice are indicated with green, red, and blue colors, respectively.

**Figure 3:**
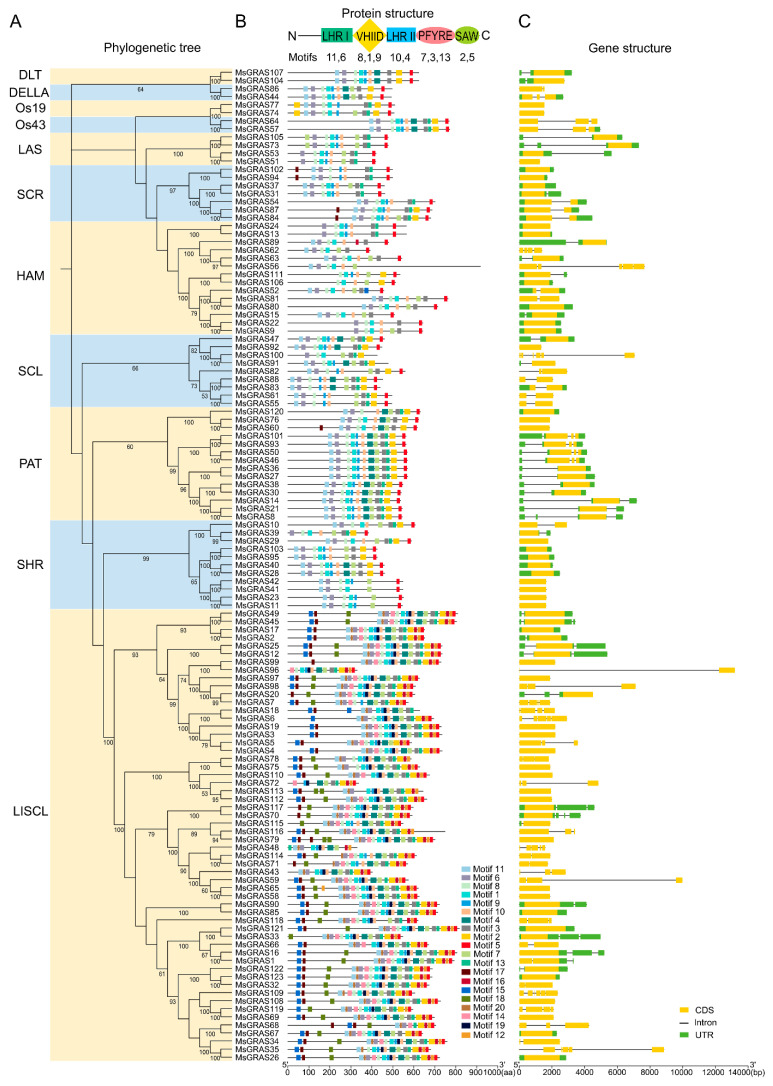
Phylogenetic relationship, motif distribution, and gene structure of MsGRAS. (**A**) Phylogenetic tree constructed based on the alignments of 123 MsGRAS full-length amino acid sequences. Bootstrap values higher than 50% are shown. (**B**) Conserved motifs in MsGRAS proteins. Each motif is shaded with colored boxes. (**C**) Gene structure of *MsGRAS*. Un-translated region (UTR), exons, and introns are indicated by green rectangles and yellow and black lines, respectively.

To reveal the phylogenetic relationship among MsGRAS members, the 123 full-length MsGRAS protein sequences together with GRAS members from Arabidopsis (33) and rice (50), were used to construct an un-rooted neighbor-joining (NJ) phylogenetic tree (Figure 2). The GRAS proteins were classified into 11 subfamilies according to the nomenclature of Arabidopsis and rice GRAS subfamilies in previous studies [31,32]. The MsGRAS proteins were divided into 10 subfamilies except for subfamily SCL4/7 (Figure 3A). The LISCL subfamily was the largest one with 56 MsGRAS members, followed by the HAM subfamily with 14 members. By contrast, the DLT, DELLA, Os19, and Os43 subfamilies were the smallest ones with only two MsGRAS members for each subfamily. It was noteworthy that the number of the MsGRAS members (56) in the LISCL subfamily was significantly expanded compared to the numbers of their counterparts in Arabidopsis (7) and rice (10).

To investigate the features of the conserved domain of MsGRAS, we used MEME to analyze the pattern of motifs in MsGRAS proteins. A total of 20 motifs were discovered in MsGRAS proteins (Figure 3B). The amino acid composition of these identified motifs is presented in Figure S2. The majority of motifs were located in the conserved C-terminus compared to the N-terminus, highlighting the functional importance of the C-terminal region. Twelve motifs were identified to correspond to the five sub-domains in the conserved C-terminal GRAS domain, namely LHRI (motifs 11 and 6), VHIID (motifs 1, 8, and 9), LHRII (motifs 4 and 10), PFYRE (motifs 3, 7, and 13), and SAW (motifs 2 and 5). Despite that the C-terminal region is highly conserved, not all MsGRAS proteins possessed all the five subdomains with corresponding motifs. For instance, MsGRAS18, MsGRAS48, and MsGRAS56 lacked the SAW domain, while the LHRII domain was absent in MsGRAS11, MsGRAS23, MsGRAS41, and MsGRAS42. Generally, the MsGRAS members classified in the same subfamily shared very common motif compositions. For example, the 12 MsGRAS members in the PAT1 subfamily shared 14 identical motifs that were arranged in the same order. The motif compositions of the MsGRAS members in the DLT, DELLA and Os43 subfamilies were identical. It was noteworthy that the N-terminus of MsGRAS proteins in the HAM subfamily was more variable compared with other subfamilies.

Furthermore, we analyzed the pattern of exon-intron distribution in *MsGRAS* genes to assess its structural diversity (Figure 3C). The results showed that 45 out of 123 genes (36.6%) were intronless genes. The pattern of the intron in terms of size and number was variable between *MsGRAS* genes. For instance, *MsGRAS96* contained an extremely large intron (12.2 kb) whereas *MsGRAS48* possessed four tiny introns with varying sizes (0.036 to 0.384 kb). Nevertheless, the pattern of the intron was highly conserved in the same *MsGRAS* subfamily. For example, 13 out of 14 PAT members contained introns with similar patterns, while 9 out of 11 SHR members lacked introns.

### 2.4. Cis-Elements in MsGRAS Promoters

**Figure 4:**
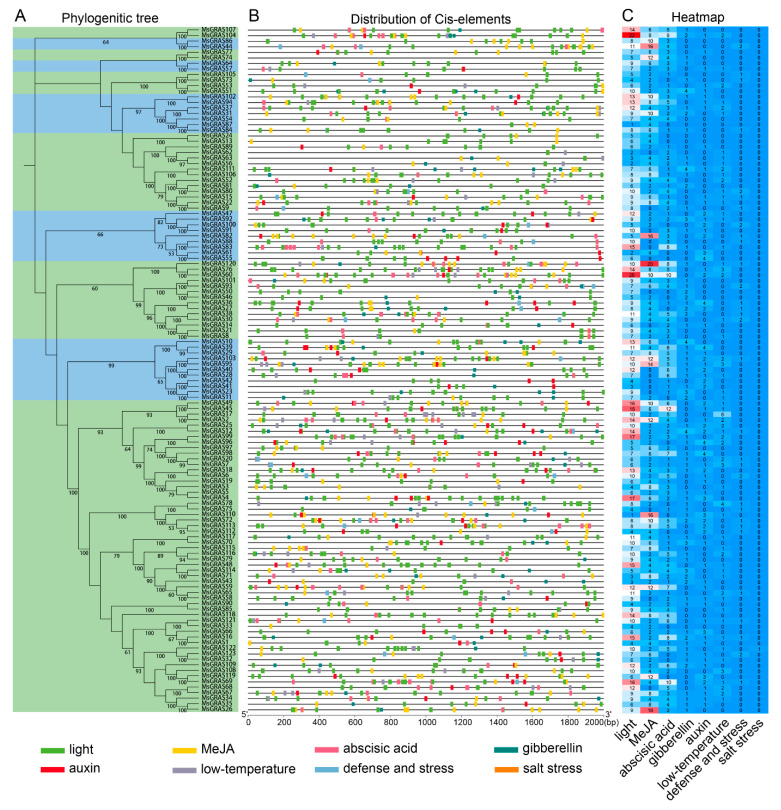
Predicted cis-elements in the promoter sequences of *MsGRAS* genes. (**A**) Phylogenetic tree of *MsGRAS*. (**B**) Distributions of cis-elements in *MsGRAS* promoters. The rectangles with different colors indicate various cis-elements. The number at the bottom indicates the 2000 bp upstream nucleotides to the transcription start codon. (**C**) Heat map showing the occurrence of cis-elements in *MsGRAS* gene promoters. The scale bar indicates the occurrence of cis-elements with red indicating a larger number and blue denoting a smaller number.

We identified the putative cis-elements within 2000 bp *MsGRAS* promoter sequences upstream of the start codon. The display of the cis-elements in *MsGRAS* promoters was arranged according to their phylogenetic relationships (Figure 4A). The results showed that the majority of cis-elements identified were associated with light, hormone, and stress responses (Figure 4B). For example, light-responsive cis-elements were present in almost all *MsGRAS* gene promoters, while phytochrome response elements were only present in *MsGRAS18* and *MsGRAS47* promoters. In addition, the position and occurrence frequency of these cis-elements in each *MsGRAS* gene varied significantly (Figure 4C). For example, cis-elements related to MeJA, light, abscisic acid, and stress responses appeared multiple times at different positions for each *MsGRAS* gene, among which the cis-elements involved in MeJA and light responses appeared most frequently. However, the position and occurrence frequency of cis-elements exhibited no substantial difference among the *MsGRAS* genes in the same subfamily (Figure 4A,C). Moreover, the paralogous gene pairs, such as *MsGRAS11*/*MsGRAS23*, *MsGRAS28*/*MsGRAS40*, *MsGRAS30*/*MsGRAS38*, *MsGRAS45*/*MsGRAS49*, *MsGRAS46*/*MsGRAS50*, and *MsGRAS91*/*MsGRAS100* possessed almost identical types of cis-elements with similar occurrence.

### 2.5. Expression Profiling of MsGRAS Genes in Salt and Alkali Stress

**Figure 5:**
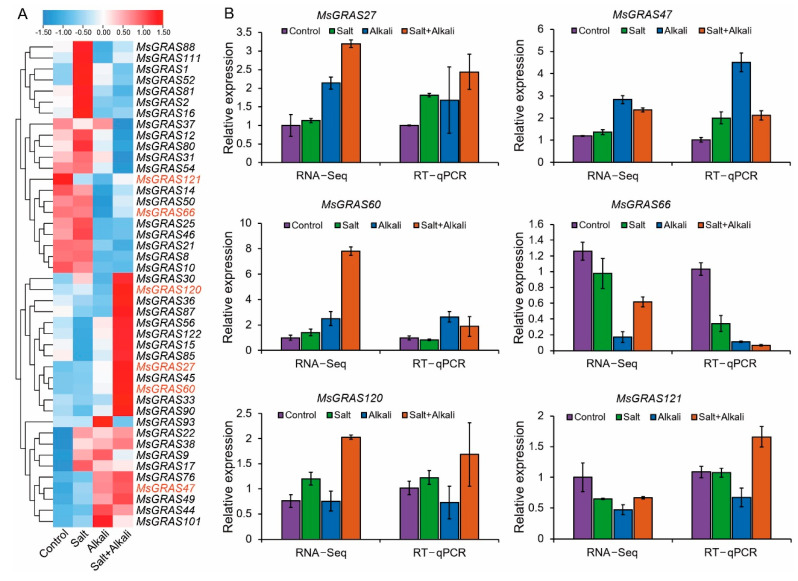
Expression profiling of *MsGRAS* genes and verification by RT-qPCR. (**A**) Expression profiling of *MsGRAS* genes under salt, alkali, and mixed salt-alkali stresses. The transcriptome sequencing was performed using Miscanthus leaves subjected to salt (0.3M NaCl + 0.3M Na_2_SO_4_), alkali (0.315M Na_2_CO_3_ + 0.315M NaHCO_3_), and salt-alkali (0.15M NaCl, 0.15M Na_2_SO_4_, 0.15M Na_2_CO_3_, and 0.15M NaHCO_3_) treatments for 6 h. The genes in red were selected for RT-qPCR verification. (**B**) Comparison of RT-qPCR and RNA-seq analyses of six *MsGRAS* gene expression in salt, alkali, and mixed salt-alkali treatments. The bars represent the standard error (SE) of three biological repeats.

To explore the expression profile of *MsGRAS* genes in response to saline or/and alkaline stress, we mined their expression profiles using the transcriptome data (unpublished). At least 3, 4, and 8 *MsGRAS* genes exhibited significantly up-regulated expression (more than two-fold change) under salt, alkali, and mixed salt-alkali stresses, respectively (Figure 5A). For example, the expression of *MsGRAS1* and *MsGRAS22* was specifically up-regulated under salt stress, while the expression of *MsGRAS47*, *MsGRAS49,* and *MsGRAS60* was specifically up-regulated under alkali and mixed salt-alkali stresses. Moreover, the expression of *MsGRAS27*, *MsGRAS90*, and *MsGRAS120* was up-regulated under mixed salt-alkali stress. In addition, the expression of *MsGRAS10*, *MsGRAS121,* and *MsGRAS66* was down-regulated under alkali stress, while the expression of *MsGRAS10* and *MsGRAS37* was down-regulated under mixed salt-alkali stress.

To verify the expression profiles of *MsGRAS* genes under salt and alkali treatments, the relative expression levels of six *MsGRAS* genes were analyzed by RT-qPCR under salt stress treatment for 8 h. Generally, the expression of *MsGRAS* genes by RT-qPCR was largely consistent with the transcriptome data (Figure 5A,B and Figure S3). For example, the expression of *MsGRAS27*, *MsGRAS47*, and *MsGRAS60* was significantly up-regulated while *MsGRAS66* and *MsGRAS121* were significantly down-regulated after the alkali stress treatment. The expression of *MsGRAS27*, *MsGRAS60* and *MsGRAS120* was increased under the mixed salt-alkali stress treatment.

**Figure 6:**
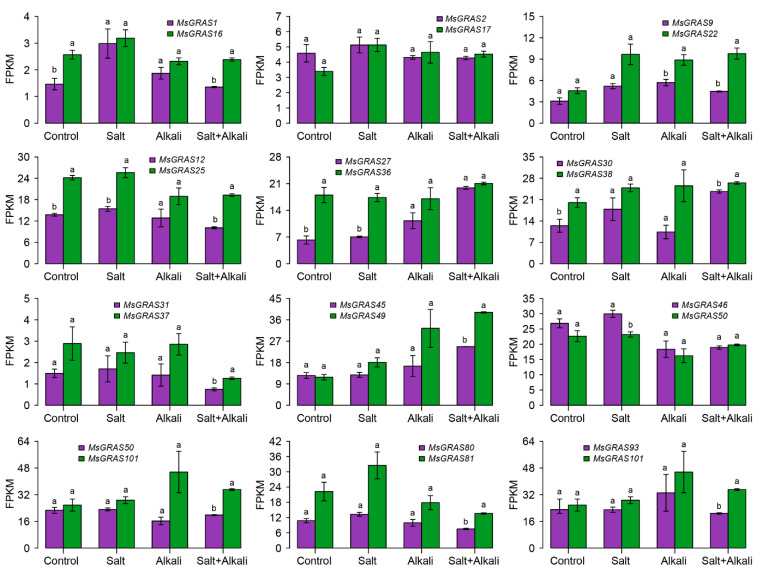
Differential expression of *MsGRAS* paralogous pairs under salt, alkali and mixed salt-alkali stresses. The same letters (a/a) mean no significant differences between paralogous genes. The different letters (a/b) represent significant differences between paralogous genes (*p* < 0.05). Error bars indicate the standard error (SE) of three biological replicates.

To investigate the expression patterns of paralogous *MsGRAS* gene pairs, we compared their expression levels under salt and/or alkali stress treatments. The results showed that the expression of several gene pairs, such as *MsGRAS1*/*MsGRAS16*, *MsGRAS12*/*MsGRAS25*, *MsGRAS27*/*MsGRAS36,* and *MsGRAS30*/*MsGRAS38*, exhibited significant differences in the absence of stress treatment (Control) (Figure 6). By contrast, the expression of most paralogous gene pairs displayed almost identical patterns under the salt, alkali, and mixed salt-alkali stress treatments. However, the expression of several paralogous gene pairs such as *MsGRAS46*/*MsGRAS93* and *MsGRAS50*/*MsGRAS101* showed divergent patterns under alkali stress treatment. Moreover, paralogous gene pairs such as *MsGRAS9*/*MsGRAS22*, *MsGRAS45*/*MsGRAS49*, *MsGRAS50*/*MsGRAS101*, and *MsGRAS93/MsGRAS101* exhibited significant expression differences after the salt and alkali stress treatments. Interestingly, gene pairs (i.e., *MsGRAS12*/*MsGRAS25* and *MsGRAS27*/*MsGRAS36)* exhibited a significant expression difference when the stress was absent whereas the difference narrowed when subjected to the alkali stress.

### 2.6. MsGRAS60 Overexpression Confers Compromised Salt and Alkali Tolerance in Arabidopsis

**Figure 7:**
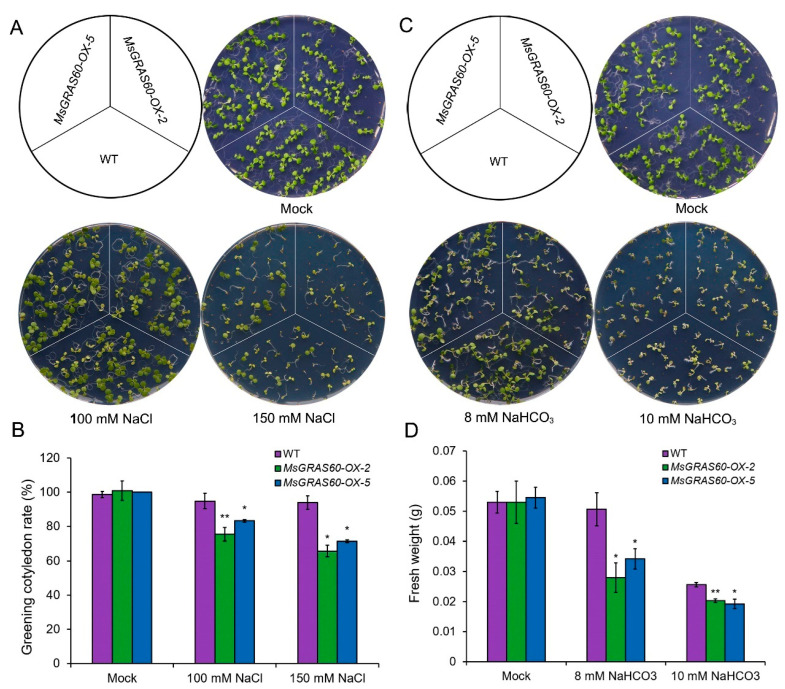
Salt and alkali stress tolerance assay of *MsGRAS60* overexpression lines. (**A**) Germination assay of WT and *MsGRAS60* overexpression lines under mock, 100 mM, and 150 mM NaCl treatments. (**B**) Quantification of greening cotyledon frequency in salt stress treatment. The greening cotyledon rate was calculated as the percentage of seedlings with green cotyledon out of the whole seedlings in three biological replicates. (**C**) Germination assay of *MsGRAS60* transgenic lines and WT seedlings under mock, 8 mM, and 10 mM NaCHO_3_ treatments (**D**) Quantification of fresh weight of seedlings under alkali stress treatment. At least 20 seedlings for each background were measured with three biological replicates. Values represent mean ± SE. Asterisks indicate significant differences between transgenic plants and WT based on Student’s *t*-test (* *p* < 0.05; ** *p* < 0.01).

The expression analysis revealed that *MsGRAS60* was up-regulated in salt and alkali stress treatments (Figure 5). We subsequently examined its functional role in response to salt and alkali stresses in transgenic *Arabidopsis*. At least 10 transgenic lines were obtained, and two representative lines (*MsGRAS60-OX-2* and *MsGRAS60-OX-5*) with higher expression levels were selected for the phenotypic analyses. Firstly, we measured the greening cotyledon rate of the wild type (WT) and two *MsGRAS60* overexpression lines under normal and salt stress conditions. There was no significant difference in the occurrence rate of greening cotyledons between the *MsGRAS60* overexpression lines and WT under normal growth conditions (Figure 7A,B). When subjected to 100 mM and 150 mM NaCl treatments, the percentage of greening cotyledons of the *MsGRAS60* overexpression lines was much lower than that of the WT (Figure 7A,B).

Furthermore, we examined the performance of the *MsGRAS60* overexpression lines and WT under normal and alkali stress conditions. Although two overexpression lines exhibited comparable germination rates to the WT under normal, 8 mM, and 10 mM NaHCO_3_ treatments, the growth of transgenic lines was significantly inhibited compared to that of the WT (Figure 7C,D). Accordingly, the average fresh weight of the overexpression lines was significantly lower than the WT under 8 mM and 10 mM NaHCO_3_ treatments (Figure 7D). These results indicated that *MsGRAS60* overexpression lines are more sensitive to salt and alkali stresses.

### 2.7. MsGRAS60 Overexpression Alleviates ABA Sensitivity in Arabidopsis

**Figure 8:**
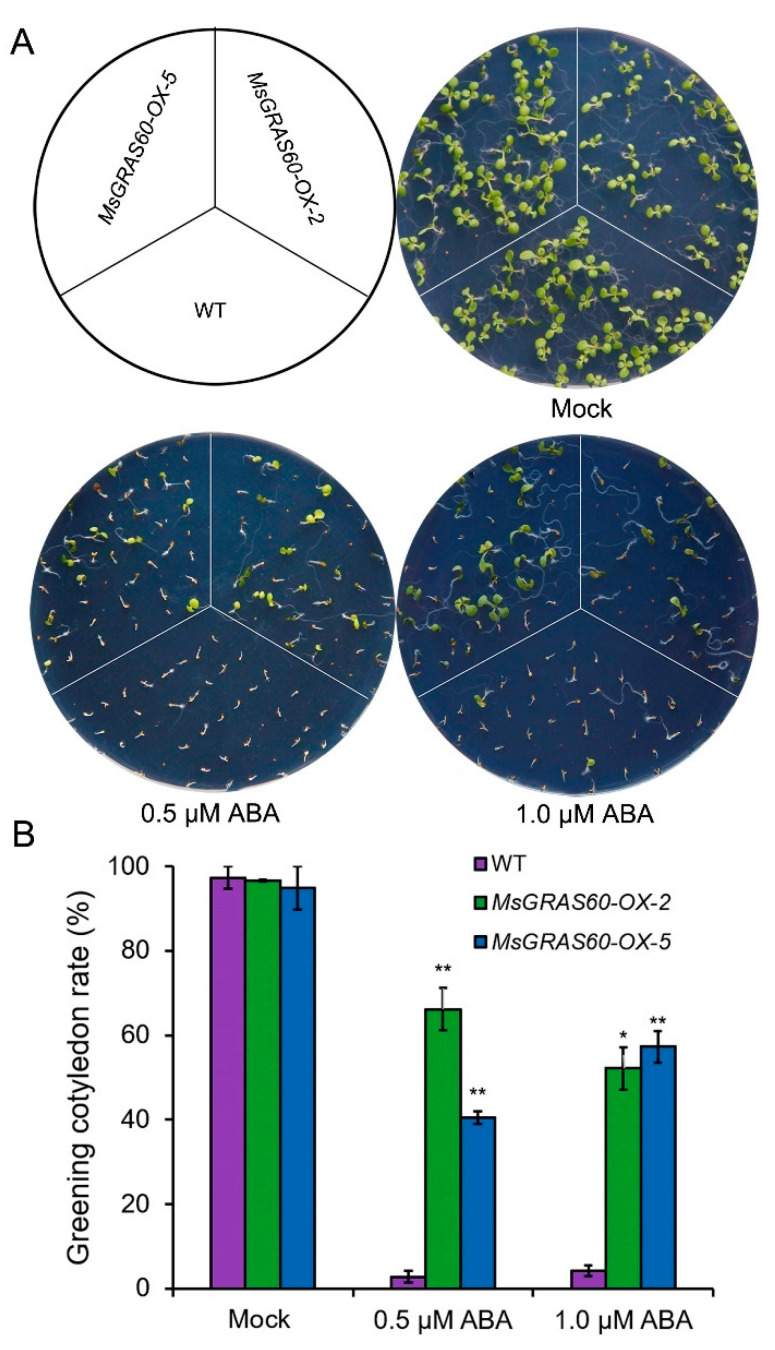
ABA sensitivity assay of *MsGRAS60* overexpression lines. (**A**) Germination assay of WT and two *MsGRAS60* overexpression lines. Seeds were sown on the 1/2 MS plate supplemented with 0, 0.5, and 1.0 μM ABA. (**B**) Statistics of greening cotyledon rates of WT and *MsGRAS60* overexpression lines on 1/2 MS plate supplemented with 0, 0.5, and 1.0 μM ABA. Values represent mean ± SE of three biological replicates. Asterisks indicate significant differences between transgenic plants and WT based on Student’s *t*-test (* *p* < 0.05; ** *p* < 0.01).

To verify if the compromised tolerance to salt and alkali stresses of *MsGRAS60* transgenic lines was associated with alterations in ABA sensitivity, we measured the greening cotyledon rate of WT and two *MsGRAS60* overexpression lines under 0.5 and 1.0 μM ABA treatments (Figure 8A). Under normal growth condition, the *MsGRAS60* overexpression lines and WT seeds exhibited comparable greening cotyledon rates (Figure 8A,B). However, when subjected to 0.5 and 1.0 μM ABA treatments, the greening cotyledon rates of the overexpression lines were significantly higher than WT (Figure 8A,B). These results suggest that *MsGRAS60* overexpression confers hyposensitivity to ABA in transgenic *Arabidopsis*.

## 3. Discussion

### 3.1. MsGRAS Gene Duplication and Phylogenetic Relationship

Through bioinformatic analysis, we identified 123 *MsGRAS* genes from the genome of *M. sinensis*. Compared to the reported *GRAS* gene family in other plant species, such as Arabidopsis (34) [31], rice (60) [31], tomato (53) [33], maize (86) [34], sorghum (81) [35], poplar (106) [31], and cotton (150) [36], the number of *GRAS* genes in *M. sinensis* was the second highest. The differences in the *GRAS* gene family among various plant species may be either attributed to the genome size of the species or resulted from gene duplication events during the evolutionary process [37]. Segmental and tandem duplications represent two major types of evolutionary patterns in plants. Segmental duplication is primarily derived from the chromosomal rearrangement that generates numerous duplicated chromosomal blocks in plant genomes [38]. The expansion of various gene families has been revealed to be attributed to segmental duplication during evolution. Our results showed that segmental duplication was mainly responsible for the expansion of *GRAS* gene family in *M. sinensis*. At least 59 genes accounting for 48% of the total *MsGRAS* genes experienced segmental duplication events (Figure 1).

Similarly, most of the plant *GRAS* gene families identified so far have been suggested to have experienced segmental duplication events, such as Arabidopsis (48%) [31], plum (89%) [39], rice (40%) [31], sorghum (31%) [33], alfalfa (37%) [40], and poplar (73%) [31]. Compared to the occurrence of segmental duplication events in other plant species, the ratio of *MsGRAS* genes experiencing segmental duplication was comparable to the other plant species. It was worth noting that *MsGRAS* genes exclusively experienced segmental duplication while no tandem duplication was detected (Table 2). The results are in agreement with a multilayered cross-species analysis of *GRAS* gene family [41]. These results imply that segmental duplication plays an indispensable role in the expansion of *GRAS* gene family. Nevertheless, the segmental duplication alone could not fully explain the large *GRAS* gene family in *M. sinensis*.

Gene duplication events during the evolution process provide an impetus for gene loss, functional divergence, and generation of novel gene functions [42]. Our results revealed 35 paralogous gene pairs derived from the segmental duplication (Table 2). Generally, paralogous pairs originating from segmental duplication may undergo different types of evolutionary fates [43]. The Ka/Ks ratios of the duplicated paralogous gene pairs of *MsGRAS* were lower than 1.0, implying that they have undergone purifying selection and experienced limited functional divergence (Table 2). Correspondingly, the two gene members in most of the paralogous pairs exhibited identical or similar salt and alkali-inducible expression patterns as revealed by the transcriptome data (Figure 6). This suggests that most of them have limited functional divergence in salt and alkali stress response in Miscanthus. However, the expression of several paralogous pairs, such as *MsGRAS44*/*MsGRAS88* and *MsGRAS48*/*MsGRAS96*, exhibited divergent differences under salt and mixed salt-alkali stress treatments (Figure 6), implying that they might have divergent functional roles in these stressed conditions. The exact roles in salt and alkali stress tolerance await further detailed functional characterization in transgenic plants.

Previous studies revealed that genomic duplication events account for the expanding of transcription factor gene families and the enhanced plant tolerance to various stressed conditions in plants [44,45]. Our study showed that several paralogous pairs such as *MsGRAS9*/*MsGRAS22*, *MsGRAS45*/*MsGRAS49*, *MsGRAS50*/*MsGRAS101*, and *MsGRAS93*/*MsGRAS101* exhibited almost identical expression levels under unstressed conditions, while displayed significantly diversified expression patterns under salt or mixed salt and alkali stress treatments. It implies that these paralogous pairs derived from segmental duplication could have undergone substantial functional divergence to deal with the adverse environments. The divergence of the paralogous pairs may help to enhance the adaptability of Miscanthus plants to cope with various unfavorable environments.

### 3.2. MsGRAS Protein Structure Characteristic and Function Prediction

GRAS proteins are characterized by a highly conserved C-terminal domain and a relatively diversified N-terminal region [16]. Our study showed that 123 MsGRAS proteins contained the conserved C-terminal domains composed of five subdomains namely LHR I, VHIID, LHR II, PFYRE, and SAW (Figure 3B), which is consistent with the previous studies of *GRAS* gene families in other plant species [46]. It was noteworthy that the number of conserved motif numbers and their arrangement among different MsGRAS subfamilies varied substantially, indicative of potential functional diversification of the MsGRAS members. For instance, the MsGRAS proteins in the LISCL subfamily contained eight motifs (motifs 12, 14, 15, 16, 17, 18, 19, and 20) that were rarely present in the other subfamilies. It remained to be clarified whether these motifs are associated with more variable biological roles for the MsGRAS members in the LISCL subfamily.

Previous studies revealed that the conserved C-terminal subdomains play an essential role in GA signaling, thus significantly affecting plant development. For example, mutations in the PFYRE and SAW motifs lead to enhanced growth in Arabidopsis [47,48,49]. Intriguingly, our results showed that several MsGRAS proteins, such as MsGRAS56 in the HAM subfamily and MsGRAS18 and MsGRAS48 in the LISCL subfamily, lacked the SAW subdomains (motif 2) in the GRAS domain (Figure 3B). Additionally, it was noteworthy that the SAW subdomain was nested in the C and N-terminal regions of the MsGRAS77 and MsGRAS74 in the Os19 subfamily. This is not consistent with the previous studies in other species, such as sorghum, castor beans, and cassava [32,35,50]. These differences in motif composition and arrangement could give rise to distinct roles of *MsGRAS* genes in Miscanthus, which warrants further investigation for their exact biological roles.

Introns play a significant role during the evolutionary progress in eukaryotes, and the evolution of intron is usually accompanied by gene segmental duplication in plants [51]. Previous studies revealed that a high percentage of intronless genes are predominant in *GRAS* gene families, such as Arabidopsis (67%) [31], rice (55%) [31], tomato (77%) [52], potato (90%) [53], maize (80%) [34], and common bean (93%) [18]. It implies that *GRAS* genes have undergone substantial intron loss events during the evolutionary process. However, our study revealed that only 45 *MsGRAS* genes are intronless, accounting for 37% of the total number (Figure 3C). The percentage of intronless genes in the Miscanthus *GRAS* gene family was much lower compared to the other species in previous studies.

Moreover, it was worth noting that the intron size and number were variable between *MsGRAS* members even from the same subfamily. For example, *MsGRAS100* had six introns with different sizes, while its close homologs *MsGRAS91* and *MsGRAS92* from the same subfamily contained zero and one intron, respectively. Furthermore, several paralogous pairs derived from segmental duplication, such as *MsGRAS9*/*MsGRAS15*, *MsGRAS80*/*MsGRAS81*, and *MsGRAS50*/*MsGRAS93*, exhibited significant differences in intron composition, size, and number (Figure 3C). It remained unclear whether the diversified intron compositions are associated with the functional divergence of *GRAS* genes in Miscanthus.

Cis-elements play a significant role in the transcriptional regulation of gene expression in response to abiotic stresses [54]. Light-responsive elements, followed by MeJA and ABA response elements, were among the most predominant cis-elements in the *MsGRAS* promoters (Figure 4). Moreover, the cis-elements associated with ABA, low-temperature, defense, and salt-stress responsive elements were frequently present in *MsGRAS* promoters. These cis-elements provided clues for the functional roles of *MsGRAS* genes in regulating the growth and response to various biotic/abiotic stresses.

### 3.3. Overexpression of MsGRAS Gene and Abiotic Stress Response

The transcriptome profiling combined with RT-qPCR analysis led to the identification of six *MsGRAS* genes, belonging to three subfamilies, involved in salt and/or alkali stress response. Among these salt and/or alkali stress-inducible *MsGRAS* genes, *MsGRAS66* and *MsGRAS121* belonged to the LISCL subfamily and *MsGRAS47* to the SCL3 subfamily. *MsGRAS27*, *MsGRAS60*, and *MsGRAS120* were classified into the PAT subfamily. The orthologs of *MsGRAS60* in Arabidopsis (*AtSCL8*) and rice (*OsGRAS10*) are involved in the regulation of salt (NaCl) stress response via an ABA-dependent signaling pathway [55]. Likewise, the expression of *MsGRAS60* was significantly up-regulated under alkali, as well as the mixed salt and alkali stress treatment (Figure 5A,B). We functionally characterized the role of *MsGRAS60* in salt and alkali stress tolerance in transgenic Arabidopsis. The results showed that overexpression of *MsGRAS60* led to hyposensitivity to ABA, and compromised salt and alkali stress tolerance (Figure 7 and Figure 8). Therefore, *MsGRAS60* acts as a negative transcriptional regulator in salt and alkali stress tolerance via an ABA-dependent manner. It can be anticipated that mutation of *MsGRAS60* via Virus-induced gene silencing (VIGS), or gene editing technique with clustered regularly interspaced short palindromic repeats (CRISPR)/CRISPR-associated protein 9 (Cas9) will lead to the enhanced salt and/or alkali stress tolerance in transgenic Miscanthus. Therefore, *MsGRAS60* holds a potential application as an ideal candidate for the genetic improvement of abiotic stress tolerance in Miscanthus.

## 4. Materials and Methods

### 4.1. The Identification of GRAS Genes in M. sinensis

Genome sequences of *M. sinensis* were downloaded from Phytozome (http://www.phytozome.net/) (accessed on 2 September 2021) and GRAS protein sequences of Arabidopsis were downloaded from the Arabidopsis Information Resource (TAIR) (https://www.arabidopsis.org/index.jsp) (accessed on 3 September 2021). The hidden Markov model (HMM) profile of GRAS domain (PF03514) was downloaded from the Pfam database (http://pfam.sanger.ac.uk/) (accessed on 3 September 2021). Local BLASTP was performed using GRAS protein sequences of Arabidopsis and HMM profile as queries against the proteome sequences of *M. sinensis* with an e-value cut-off at 1 × 10^−5^, respectively. The candidate sequences were filtered by an online conserved domain (CD) search at NCBI to confirm the GRAS domain. For genes with several alternative splicing variants, only the longest sequence was retained. The closest *MsGRAS* orthologs to Arabidopsis were predicted by BLASTP searching against the Arabidopsis proteome sequences (V11.0) (https://www.arabidopsis.org/) (accessed on 21 September 2021).

### 4.2. Phylogenetic Analysis

The neighbor-joining (NJ) tree was constructed with MEGA software (Version 11, Mega Limited, Auckland, New Zealand) [56] using the full-length sequences of MsGRAS proteins. The bootstrap analysis was carried out with 1000 replicates. Only support values higher than 50% were shown on the clades. The other parameters were adopted as default.

### 4.3. Gene Structure and Conserved Motif Analysis

The exon and intron structure of each *MsGRAS* gene was displayed using the TBtools software (Version 1.098769, South China Agricultural University, Guangzhou, China) [57]. Conserved motifs of MsGRAS protein were identified using the online MEME program (Version 5.4.1) (http://meme-suite.org/tools/meme) (accessed on 18 December 2021). The maximum motif number was set as 20, and the other parameters as default. The motifs were matched to the conserved domain (LHRI, VHIID, LHRII, PFYRE, and SAW) by the global alignments within MsGRAS proteins.

### 4.4. Chromosomal Mapping and Segmental Duplication Analysis

*MsGRAS* genes were in silico mapped onto chromosomes according to the genome annotation information obtained from Phytozome (http://www.phytozome.net/) (accessed on 2 September 2021). The substitution rates of synonymous (Ks) and non-synonymous (Ka) sites for each paralogous pair were calculated by KaKs_Calculator software (Version 3.0, Chinese Academy of Sciences, Beijing, China) [58]. The duplicated *MsGRAS* genes on segmental fragments were illustrated using the TBtools software [57]. The approximate divergence date (T) of paralogous pairs was calculated using the formula T = Ks/2λ (λ equals 2.1 × 10^−8^ for Miscanthus) [14].

### 4.5. Promoter Cis-Element Analysis

Promoter sequences located 2000 bp upstream of the ATG of *MsGRAS* genes were retrieved from Phytozome (http://www.phytozome.net/) (accessed on 29 December 2021). The cis-elements in promoter sequences were analyzed using the online Plantcare tool (http://bioinformatics.psb.ugent.be/webtools/plantcare/html/) (accessed on 30 December 2021).

### 4.6. Plant Materials and Abiotic Stress Treatment

The *M. sinensis* plants were clonally propagated and transplanted into potting soil with comparable sizes. The soil was mainly composed of coconut bran, vermiculite, perlite, humus, and peat (3:1:2:2:12, *w*/*w*). After being grown for 2 months, the plants were irrigated with salt (0.3 M NaCl and 0.3 M Na_2_SO_4_), alkali (0.315 M Na_2_CO_3_ and 0.315 M NaHCO_3_), and mixed salt-alkali (0.15 M NaCl, 0.15 M Na_2_SO_4_, 0.15 M Na_2_CO_3_, and 0.15 M NaHCO_3_) solutions, respectively. For RNA-seq, the top three fully expanded leaves from the bottom were collected at 0 and 6 h of each stress treatment. The transcriptome analysis was performed at the BGI Tech Co., Ltd. (Beijing, China) (unpublished data). For RT-qPCR analysis, the top three fully expanded leaves were harvested at 8 h of each stress treatment. Leaf samples were flash-frozen in liquid nitrogen and stored at −80 °C. Each experiment was carried out with three replicates.

### 4.7. RNA Isolation and RT-qPCR

Total RNA was isolated from leaves using the TRIZOL reagent following the procedure of the manufacturer. The first strand of cDNA was synthesized with 1.0 μg RNA using cDNA Synthesis SuperMix (TransGen). The real-time quantitative PCR (RT-qPCR) analysis was performed on a StepOne Plus (ABI) system using SYBR Premix ExTaq (TaKaRa). PCR reactions were carried out with triplicate. The relative expression of target genes was normalized to the internal reference gene *ACTIN* (*Unigene33024*) [59] using the 2^−ΔΔCT^ method. Primers are listed in Table S1.

### 4.8. Generation of MsGRAS60 Overexpression Lines in Arabidopsis

The coding region of *MsGRAS60* was amplified by PCR and fused with six copies of the MYC tag at the N-terminus under the control of *CaMV35S* promoter in the modified PBI121-MYC vector [60]. Arabidopsis Columbia-0 (WT) plants by *Agrobacterium tumefaciens* mediated transformation [61]. Positive transgenic lines were screened in 1/2 MS plates containing the kanamycin (50 mg mL^−1^). Two homozygous transgenic Arabidopsis lines with higher expression levels of *MsGRAS60* were selected for phenotypic analysis under abiotic stress treatments.

### 4.9. Germination Rate and Fresh Weight Measurements under Salt and Alkali Stresses

Seeds of *MsGRAS60* transgenic lines and WT were sown on 1/2 MS agar plate supplemented with different concentrations of NaCl (0, 100, and 150 mM), NaHCO_3_ (0, 8, and 10 mM), and ABA (0, 0.5, and 1.0 μM). After stratification at 4 °C for 3 d, seeds were germinated in a growth chamber (21 °C, relative humidity 60%) under a long-day photoperiod (16-h light/8-h dark). The percentage of seedlings with greening cotyledons out of the total seedlings was recorded at 7 d. The fresh weight of seedlings under alkali stress treatment was measured at 10 d. The assays were performed with three independent replicates with at least 20 seedlings.

## 5. Conclusions

In this study, we carried out a comprehensive bioinformatics analysis of the *GRAS* gene family in *M. sinensis*. A total of 123 *MsGRAS* genes were identified, which were phylogenetically classified into ten subfamilies. Segmental duplication predominately contributed to the expansion of *MsGRAS* gene family. Approximately 12% of *MsGRAS* genes showed inducible expression patterns under salt, alkali, and mixed salt-alkali stresses. The 35 paralogous pairs derived from segmental duplication underwent purifying selection and exhibited substantial divergences in salt and alkali stress response. Additionally, the functional role of *MsGRAS60* in salt and alkali stress tolerance was analyzed in transgenic Arabidopsis. It acted as a negative regulator of salt and alkali stress tolerance via an ABA-dependent signaling pathway. Our results paved the way for future functional studies to unravel the roles of *GRAS* genes in abiotic stress response in Miscanthus.

## Figures and Tables

**Table 1:** List of *MsGRAS* genes and their closest orthologs to Arabidopsis.

No.	Gene Symbol	Gene Loci	Arabidopsis Ortholog Loci	Arabidopsis Ortholog Symbol	Score	E-Value
1	*MsGRAS1*	Misin01G020500.1	AT2G37650.1		421	1 × 10^−136^
2	*MsGRAS2*	Misin01G025400.1	AT1G07530.1	GRAS2/SCL14	499	7 × 10^−162^
3	*MsGRAS3*	Misin01G152800.1	AT2G37650.1		427	2 × 10^−139^
4	*MsGRAS4*	Misin01G152900.1	AT2G37650.1		429	2 × 10^−140^
5	*MsGRAS5*	Misin01G153100.1	AT2G37650.1		307	3 × 10^−95^
6	*MsGRAS6*	Misin01G153200.1	AT2G29065.1		390	5 × 10^−127^
7	*MsGRAS7*	Misin01G161800.1	AT2G37650.1		397	3 × 10^−130^
8	*MsGRAS8*	Misin01G246900.1	AT5G48150.1	PAT1	566	0
9	*MsGRAS9*	Misin01G287600.1	AT4G00150.1	HAM3/LOM3	271	7 × 10^−83^
10	*MsGRAS10*	Misin01G310300.1	AT4G37650.1	SHR/SGR7	340	5 × 10^−110^
11	*MsGRAS11*	Misin01G323700.1	AT3G13840.1		234	4 × 10^−70^
12	*MsGRAS12*	Misin01G403600.1	AT2G37650.1		545	0
13	*MsGRAS13*	Misin01G408100.1	AT4G08250.1		297	2 × 10^−94^
14	*MsGRAS14*	Misin01G449800.1	AT5G48150.1	PAT1	523	0
15	*MsGRAS15*	Misin01G513100.1	AT4G00150.1	HAM3/LOM3	244	4 × 10^−74^
16	*MsGRAS16*	Misin02G001900.1	AT1G07530.1	GRAS2/SCL14	409	3 × 10^−131^
17	*MsGRAS17*	Misin02G014100.1	AT1G07530.1	GRAS2/SCL14	496	7 × 10^−167^
18	*MsGRAS18*	Misin02G151300.1	AT2G29060.1		324	6 × 10^−102^
19	*MsGRAS19*	Misin02G151700.1	AT2G37650.1		424	2 × 10^−138^
20	*MsGRAS20*	Misin02G158600.1	AT2G37650.1		457	7 × 10^−153^
21	*MsGRAS21*	Misin02G234500.1	AT5G48150.1	PAT1	576	0
22	*MsGRAS22*	Misin02G277200.1	AT4G00150.1	HAM3/LOM3	258	1 × 10^−77^
23	*MsGRAS23*	Misin02G312300.1	AT3G13840.1		239	3 × 10^−72^
24	*MsGRAS24*	Misin02G400500.1	AT4G08250.1		295	1 × 10^−93^
25	*MsGRAS25*	Misin02G410600.1	AT2G37650.1		542	0
26	*MsGRAS26*	Misin03G277400.1	AT1G07530.1	GRAS2/SCL14	423	1 × 10^−137^
27	*MsGRAS27*	Misin03G288500.1	AT5G48150.1	PAT1	519	0
28	*MsGRAS28*	Misin03G304000.1	AT3G49950.1		422	2 × 10^−145^
29	*MsGRAS29*	Misin03G306000.1	AT4G37650.1	SHR/SGR7/	410	2 × 10^−137^
30	*MsGRAS30*	Misin03G309100.1	AT1G50600.1	SCL5	525	0
31	*MsGRAS31*	Misin03G322400.1	AT5G41920.1	AtSCL23/SCL23	428	3 × 10^−148^
32	*MsGRAS32*	Misin04G284300.1	AT1G07530.1	GRAS2/SCL14	421	4 × 10^−137^
33	*MsGRAS33*	Misin04G284400.1	AT2G37650.1		442	4 × 10^−148^
34	*MsGRAS34*	Misin04G299900.1	AT2G37650.1		442	3 × 10^−145^
35	*MsGRAS35*	Misin04G300000.1	AT2G37650.1		357	1 × 10^−113^
36	*MsGRAS36*	Misin04G313500.1	AT5G48150.1	PAT1	517	4 × 10^−180^
37	*MsGRAS37*	Misin04G325100.1	AT5G41920.1	SCL23	432	2 × 10^−149^
38	*MsGRAS38*	Misin04G335700.1	AT5G48150.1	PAT1	524	0
39	*MsGRAS39*	Misin04G338800.1	AT4G37650.1	SHR/SGR7	393	1 × 10^−133^
40	*MsGRAS40*	Misin04G340300.1	AT3G49950.1		427	2 × 10^−147^
41	*MsGRAS41*	Misin04G350600.1	AT3G13840.1		253	2 × 10^−77^
42	*MsGRAS42*	Misin04G351200.1	AT3G13840.1		262	9 × 10^−81^
43	*MsGRAS43*	Misin05G054400.1	AT2G37650.1		298	2 × 10^−94^
44	*MsGRAS44*	Misin05G234500.1	AT1G14920.1	RGA2/GAI	307	1 × 10^−98^
45	*MsGRAS45*	Misin05G342400.1	AT2G37650.1		525	2 × 10^−176^
46	*MsGRAS46*	Misin05G352700.1	AT1G21450.1	SCL1	556	0
47	*MsGRAS47*	Misin05G394400.1	AT1G50420.1	SCL3	378	3 × 10^−127^
48	*MsGRAS48*	Misin05G411500.1	AT2G37650.1		201	8 × 10^−59^
49	*MsGRAS49*	Misin06G319300.1	AT2G37650.1		527	2 × 10^−177^
50	*MsGRAS50*	Misin06G347800.1	AT1G21450.1	SCL1	550	0
51	*MsGRAS51*	Misin07G079700.1	AT1G55580.1	SCL18/LAS	245	2 × 10^−76^
52	*MsGRAS52*	Misin07G432500.1	AT4G00150.1	HAM3/LOM3	261	7 × 10^−81^
53	*MsGRAS53*	Misin08G098200.1	AT1G55580.1	SCL18/LAS	228	5 × 10^−70^
54	*MsGRAS54*	Misin09G001300.1	AT3G54220.1	SGR1/SCR	553	0
55	*MsGRAS55*	Misin09G017500.1	AT1G50420.1	SCL3	264	1 × 10^−82^
56	*MsGRAS56*	Misin09G032300.1	AT5G41920.1	SCL23	130	4 × 10^−32^
57	*MsGRAS57*	Misin09G129300.1	AT1G66350.1	RGL1	312	2 × 10^−97^
58	*MsGRAS58*	Misin09G205000.1	AT2G37650.1		333	5 × 10^−105^
59	*MsGRAS59*	Misin09G205200.1	AT2G29060.1		268	2 × 10^−81^
60	*MsGRAS60*	Misin10G000200.1	AT5G52510.1	SCL8	249	7 × 10^−74^
61	*MsGRAS61*	Misin10G015400.1	AT1G50420.1	SCL3	254	1 × 10^−78^
62	*MsGRAS62*	Misin10G037000.1	AT4G08250.1		115	4 × 10^−28^
63	*MsGRAS63*	Misin10G037100.1	AT5G41920.1	SCL23	152	6 × 10^−41^
64	*MsGRAS64*	Misin10G088000.1	AT1G66350.1	RGL1/RGL	307	1 × 10^−95^
65	*MsGRAS65*	Misin10G180800.1	AT2G37650.1		317	7 × 10^−99^
66	*MsGRAS66*	Misin10G200900.1	AT2G37650.1		436	6 × 10^−144^
67	*MsGRAS67*	Misin10G201300.1	AT2G29060.1		430	1 × 10^−142^
68	*MsGRAS68*	Misin10G201500.1	AT2G37650.1		130	2.68 × 10^−31^
69	*MsGRAS69*	Misin10G201800.1	AT1G07530.1	GRAS2/SCL14	434	3.88 × 10^−142^
70	*MsGRAS70*	Misin10G202000.1	AT1G07530.1	GRAS2/SCL14	399	3.77 × 10^−130^
71	*MsGRAS71*	Misin10G202100.1	AT1G07530.1	GRAS2/SCL14	303	1.14 × 10^−93^
72	*MsGRAS72*	Misin10G202300.1	AT2G37650.1		242	4.29 × 10^−74^
73	*MsGRAS73*	Misin11G037100.1	AT1G55580.1	SCL18/LAS	225	2.42 × 10^−68^
74	*MsGRAS74*	Misin11G080600.1	AT5G41920.1	SCL23	120	9.17 × 10^−30^
75	*MsGRAS75*	Misin11G092500.1	AT2G29065.1		333	6.82 × 10^−106^
76	*MsGRAS76*	Misin11G188000.1	AT5G52510.1	SCL8	261	3 × 10^−78^
77	*MsGRAS77*	Misin12G082800.1	AT3G03450.1	RGL2	129	3.96 × 10^−32^
78	*MsGRAS78*	Misin12G093700.1	AT2G37650.1		328	1.11 × 10^−103^
79	*MsGRAS79*	Misin12G093800.1	AT2G37650.1		335	6.9 × 10^−105^
80	*MsGRAS80*	Misin12G179500.1	AT4G00150.1	HAM3/LOM3	286	5.04 × 10^−88^
81	*MsGRAS81*	Misin12G181200.1	AT4G00150.1	HAM3/LOM3	286	3.27 × 10^−87^
82	*MsGRAS82*	Misin14G012600.1	AT1G50420.1	SCL3	305	1 × 10^−97^
83	*MsGRAS83*	Misin14G013500.1	AT1G50420.1	SCL3	270	1.33 × 10^−85^
84	*MsGRAS84*	Misin14G050000.1	AT3G54220.1	SCR/SGR1	524	6.24 × 10^−179^
85	*MsGRAS85*	Misin14G112500.1	AT2G37650.1		459	1.56 × 10^−152^
86	*MsGRAS86*	Misin14G164000.1	AT2G01570.1	RGA/RGA1/RGA24	246	9.53 × 10^−75^
87	*MsGRAS87*	Misin15G009500.1	AT3G54220.1	SCR/SGR1	553	0
88	*MsGRAS88*	Misin15G025900.1	AT1G50420.1	SCL3	266	6.14 × 10^−84^
89	*MsGRAS89*	Misin15G070400.1	AT4G08250.1		116	6.37 × 10^−28^
90	*MsGRAS90*	Misin15G148100.1	AT2G37650.1		461	4.96 × 10^−153^
91	*MsGRAS91*	Misin16G120300.1	AT1G50420.1	SCL3	164	6.3 × 10^−45^
92	*MsGRAS92*	Misin16G120400.1	AT1G50420.1	SCL3	223	9.28 × 10^−68^
93	*MsGRAS93*	Misin16G145600.1	AT1G21450.1	SCL1	523	0
94	*MsGRAS94*	Misin16G178000.1	AT3G54220.1	SCR/SGR1	269	7.51 × 10^−83^
95	*MsGRAS95*	Misin16G190800.1	AT3G49950.1		293	8.04 × 10^−96^
96	*MsGRAS96*	Misin17G020200.1	AT2G37650.1		350	1.88 × 10^−115^
97	*MsGRAS97*	Misin17G020300.1	AT2G37650.1		479	3.77 × 10^−161^
98	*MsGRAS98*	Misin17G020400.1	AT2G37650.1		429	2.77 × 10^−142^
99	*MsGRAS99*	Misin17G049900.1	AT2G37650.1		409	1.46 × 10^−132^
100	*MsGRAS100*	Misin17G120700.1	AT1G50420.1	SCL3	130	4.04 × 10^−33^
101	*MsGRAS101*	Misin17G146900.1	AT1G21450.1	SCL1	541	0
102	*MsGRAS102*	Misin17G169300.1	AT3G54220.1	SCR/SGR1	274	9.87 × 10^−85^
103	*MsGRAS103*	Misin17G191000.1	AT3G49950.1		297	2.89 × 10^−97^
104	*MsGRAS104*	Misin18G011200.1	AT1G63100.1	SCL28	374	1.49 × 10^−121^
105	*MsGRAS105*	Misin18G187000.1	AT1G55580.1	SCL18/LAS	225	2.17 × 10^−68^
106	*MsGRAS106*	Misin19G008400.1	AT4G00150.1	HAM3/LOM3	242	2.74 × 10^−73^
107	*MsGRAS107*	Misin19G015600.1	AT1G63100.1	SCL28	375	1.19 × 10^−121^
108	*MsGRAS108*	MisinT086900.1	AT4G00150.1	HAM3/LOM3	233	1.74 × 10^−69^
109	*MsGRAS109*	MisinT072400.1	AT1G07530.1	GRAS2/SCL14	430	3.89 × 10^−140^
110	*MsGRAS110*	MisinT072600.1	AT2G37650.1		323	2.94 × 10^−101^
111	*MsGRAS111*	MisinT074500.1	AT2G37650.1		393	2.46 × 10^−127^
112	*MsGRAS112*	MisinT131300.1	AT2G37650.1		375	9.91 × 10^−121^
113	*MsGRAS113*	MisinT131400.1	AT2G29065.1		305	5.61 × 10^−95^
114	*MsGRAS114*	MisinT131700.1	AT1G07530.1	GRAS2/SCL14	310	7.58 × 10^−96^
115	*MsGRAS115*	MisinT131900.1	AT2G29060.1		347	2.12 × 10^−111^
116	*MsGRAS116*	MisinT132000.1	AT2G37650.1		328	1.36 × 10^−101^
117	*MsGRAS117*	MisinT132100.1	AT1G07530.1	GRAS2/SCL14	400	1.09 × 10^−130^
118	*MsGRAS118*	MisinT132200.1	AT2G37650.1		294	3.81 × 10^−90^
119	*MsGRAS119*	MisinT132300.1	AT1G07530.1	GRAS2/SCL14	342	2.5 × 10^−108^
120	*MsGRAS120*	MisinT322700.1	AT5G52510.1	SCL8	258	4.98 × 10^−77^
121	*MsGRAS121*	MisinT390500.1	AT1G07530.1	GRAS2/SCL14	453	4.6 × 10^−148^
122	*MsGRAS122*	MisinT390800.1	AT2G37650.1		446	8.22 × 10^−148^
123	*MsGRAS123*	MisinT390900.1	AT1G07530.1	GRAS2/SCL14	437	2.07 × 10^−143^

**Table 2:** Ka/Ks ratio of paralogous pairs of *MsGRAS* genes.

No.	Locus 1	Locus 2	Protein Identity (%)	Ka	Ks	Ka/Ks	Duplication Type	Purify Selection	Divergence Time (Mya)
1	*MsGRAS1*	*MsGRAS16*	93.39	0.035	0.089	0.392	Segmental	Yes	2.12
2	*MsGRAS2*	*MsGRAS17*	97.39	0.013	0.047	0.280	Segmental	Yes	1.12
3	*MsGRAS3*	*MsGRAS18*	71.10	0.169	0.288	0.588	Segmental	Yes	6.85
4	*MsGRAS9*	*MsGRAS15*	59.68	0.272	2.855	0.095	Segmental	Yes	67.97
5	*MsGRAS9*	*MsGRAS22*	95.02	0.019	0.083	0.234	Segmental	Yes	1.97
6	*MsGRAS10*	*MsGRAS39*	85.31	0.075	1.661	0.045	Segmental	Yes	39.54
7	*MsGRAS11*	*MsGRAS23*	95.60	0.012	0.161	0.074	Segmental	Yes	3.84
8	*MsGRAS11*	*MsGRAS42*	61.64	0.231	2.591	0.089	Segmental	Yes	61.68
9	*MsGRAS12*	*MsGRAS25*	98.09	0.010	0.028	0.341	Segmental	Yes	0.67
10	*MsGRAS13*	*MsGRAS24*	97.16	0.010	0.071	0.136	Segmental	Yes	1.68
11	*MsGRAS15*	*MsGRAS22*	65.69	0.275	3.108	0.089	Segmental	Yes	73.99
12	*MsGRAS23*	*MsGRAS41*	62.29	0.231	3.089	0.075	Segmental	Yes	73.56
13	*MsGRAS27*	*MsGRAS36*	97.72	0.011	0.061	0.176	Segmental	Yes	1.45
14	*MsGRAS28*	*MsGRAS40*	97.82	0.006	0.230	0.026	Segmental	Yes	5.47
15	*MsGRAS29*	*MsGRAS39*	98.44	0.005	0.110	0.049	Segmental	Yes	2.61
16	*MsGRAS30*	*MsGRAS38*	95.73	0.022	0.109	0.200	Segmental	Yes	2.59
17	*MsGRAS31*	*MsGRAS37*	96.72	0.011	0.147	0.076	Segmental	Yes	3.49
18	*MsGRAS45*	*MsGRAS49*	98.25	0.010	0.028	0.360	Segmental	Yes	0.67
19	*MsGRAS46*	*MsGRAS50*	97.19	0.013	0.041	0.310	Segmental	Yes	0.97
20	*MsGRAS46*	*MsGRAS93*	73.04	0.158	1.327	0.119	Segmental	Yes	31.60
21	*MsGRAS50*	*MsGRAS93*	73.04	0.156	1.350	0.116	Segmental	Yes	32.14
22	*MsGRAS50*	*MsGRAS101*	72.31	0.158	1.383	0.114	Segmental	Yes	32.92
23	*MsGRAS51*	*MsGRAS53*	93.48	0.023	0.234	0.098	Segmental	Yes	5.57
24	*MsGRAS51*	*MsGRAS105*	63.73	0.200	4.361	0.046	Segmental	Yes	103.83
25	*MsGRAS52*	*MsGRAS80*	67.61	0.220	1.172	0.187	Segmental	Yes	27.91
26	*MsGRAS52*	*MsGRAS81*	77.60	0.225	1.152	0.195	Segmental	Yes	27.42
27	*MsGRAS57*	*MsGRAS64*	95.31	0.016	0.164	0.095	Segmental	Yes	3.92
28	*MsGRAS74*	*MsGRAS77*	91.53	0.038	0.143	0.264	Segmental	Yes	3.40
29	*MsGRAS75*	*MsGRAS78*	84.44	0.073	0.221	0.332	Segmental	Yes	5.26
30	*MsGRAS80*	*MsGRAS81*	97.61	0.011	0.060	0.185	Segmental	Yes	1.44
31	*MsGRAS91*	*MsGRAS100*	88.21	0.053	0.140	0.379	Segmental	Yes	3.32
32	*MsGRAS93*	*MsGRAS101*	96.80	0.015	0.058	0.266	Segmental	Yes	1.38
33	*MsGRAS94*	*MsGRAS102*	97.59	0.009	0.048	0.195	Segmental	Yes	1.15
34	*MsGRAS95*	*MsGRAS103*	93.87	0.018	0.181	0.098	Segmental	Yes	4.30
35	*MsGRAS104*	*MsGRAS107*	97.27	0.010	0.049	0.199	Segmental	Yes	1.16

## Data Availability

All the data in this study are included in this published article.

## Supplementary Materials

The following supporting information can be downloaded at: https://www.mdpi.com/article/10.3390/ijms232314521/s1, Figure S1. Chromosomal location of *MsGRAS* genes; Figure S2. Conserved motifs in MsGRAS proteins; Figure S3. Correlation analysis between the RNA-Seq and RT-qPCR data; Table S1. Primer sequences used for RT-qPCR analysis.
