# Supplementary material for: Genome-Wide Characterization and Expression Profiling of the GRAS Gene Family in Salt and Alkali Stresses in Miscanthus sinensis
Source: Int J Mol Sci. 2022 Nov 22;23(23):14521. doi: 10.3390/ijms232314521 (PMC9737823; doi:10.3390/ijms232314521)
Supplement: Supplementary file 1 [file ijms-23-14521-s001.zip › ijms-1973028-supplementary.pdf]

# Supplementary Materials

## Genome-wide characterization and expression profiling of the *GRAS* gene family in salt and alkali stresses in *Miscanthus sinensis*

Xuhong Zhao [123], Yan Xu [123], Guo He [123], Kang He [123], Liang Xiao [4], Ruibo Hu [123,*] and Shengjun Li [123,*]

[1] CAS Key Laboratory of Biofuels, Shandong Provincial Key Laboratory of Energy Genetics, Qingdao Institute of Bioenergy and Bioprocess Technology, Chinese Academy of Sciences, Qingdao 266101, PR China

[2] Shandong Energy Institute, Qingdao 266101, PR China

[3] Qingdao New Energy Shandong Laboratory, Qingdao 266101, PR China

[4] College of Bioscience and Technology, Hunan Agricultural University, Changsha 410128, PR China

[*]Corresponding author:

E-mail: hurb@qibebt.ac.cn; li_sj@qibebt.ac.cn

Phone: +86-0532-80662731

Fax: +86-0532-80662778

**Figure S1.** Chromosomal location of *MsGRAS* genes.

**Figure S2.** Conserved motifs in MsGRAS proteins.

**Figure S3**. Correlation analysis between the RNA-Seq and RT-qPCR data.

**Table S1.** Primer sequences used for RT-qPCR analysis.

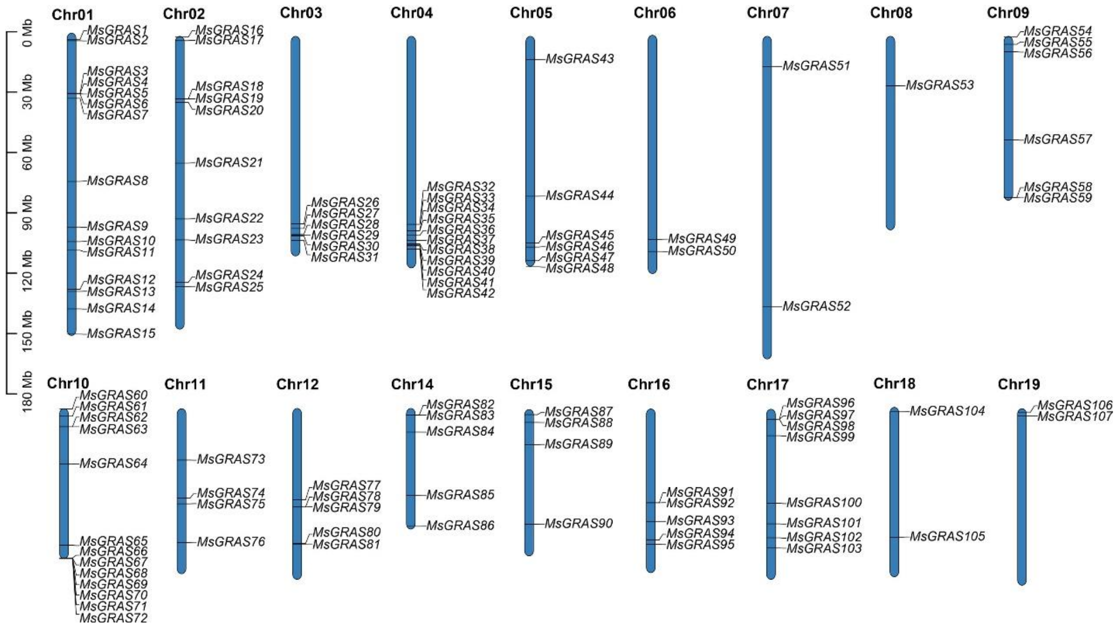


**Figure S1.** Chromosomal location of *MsGRAS* genes.

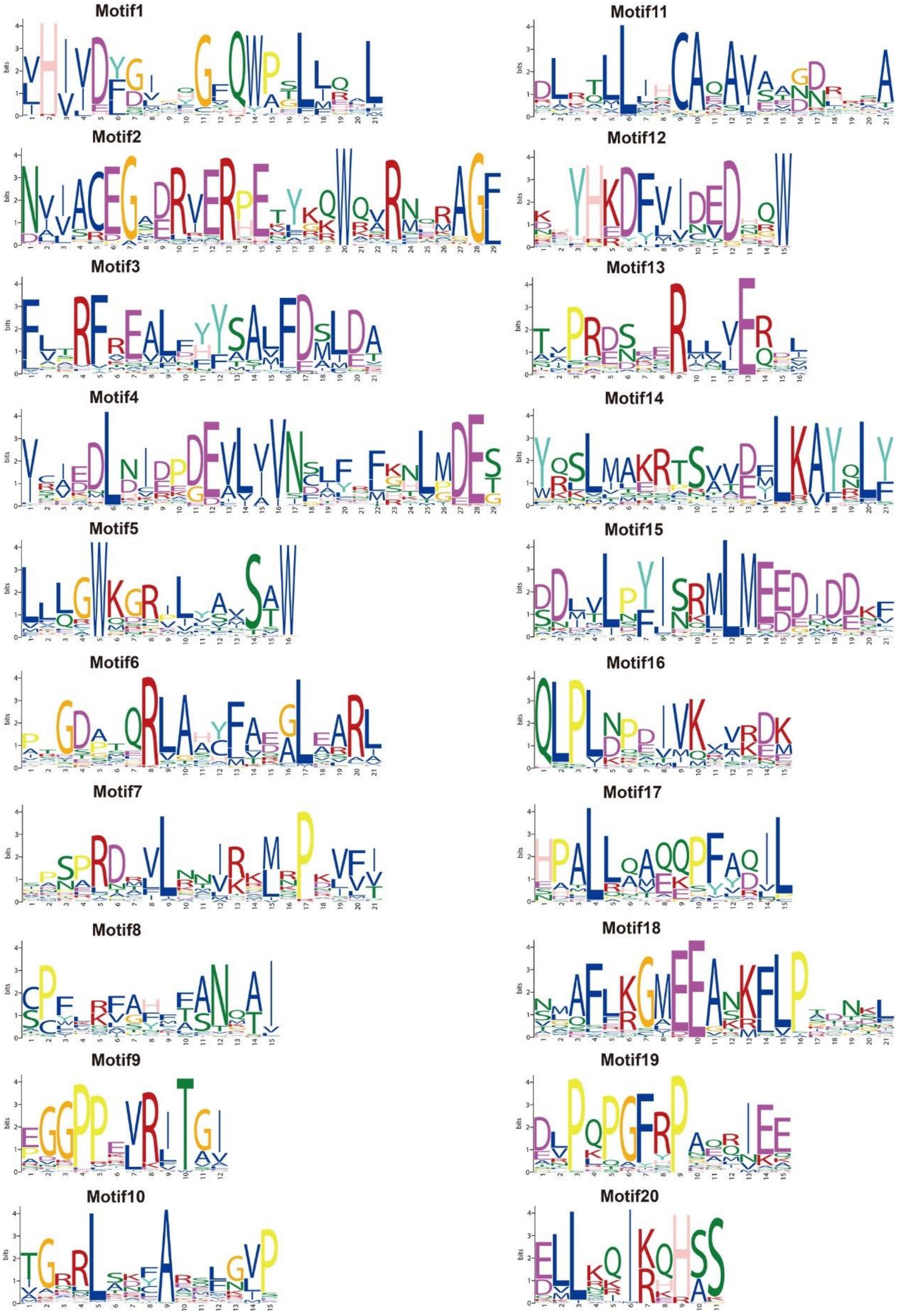


**Figure S2.** Conserved motifs in MsGRAS proteins.

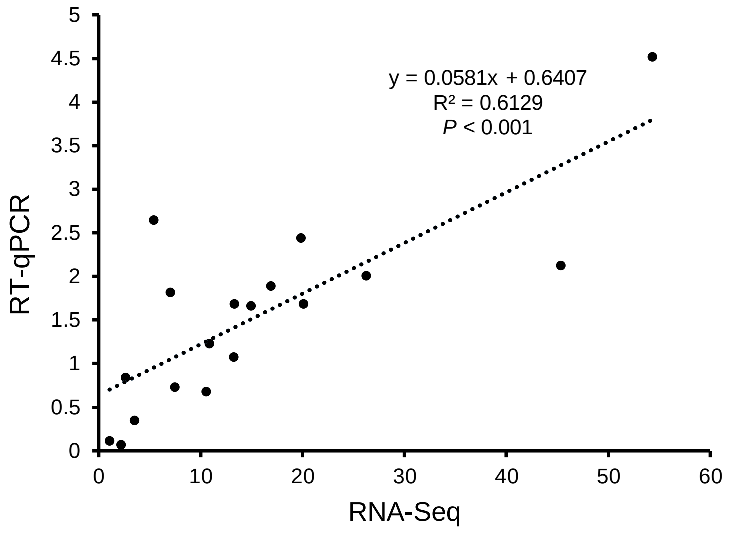


**Figure S3.** Correlation analysis between the RNA-Seq and RT-qPCR data.

**Table S1.** Primer sequences used for RT-qPCR analysis.

| **Gene Name** | **Forward primer (5' to 3')** | **Reverse primer (5' to 3')** |
|---|---|---|
| *MsGRAS1* | GTTGGCGTGCCATTCAAGTT | CCGGATGTTGTTGAGGACCA |
| *MsGRAS27* | GAGAGCCAATCCAGCGGTTA | CTCTCTGGTTCACGGCACTT |
| *MsGRAS47* | ACGTGCCGTTCCAGTTCAA | CTGCAGGTTAGAGGTGACGG |
| *MsGRAS49* | GTCATTCGCGTCATCCAACG | CGCAAAATCGGTGAAGCCAA |
| *MsGRAS60* | CAGGACATCTCACCGTGCTT | GCGAGGTACTGGATGAGAGC |
| *MsGRAS66* | AGAAACCGGTGCTAGTGAGC | CGCAATGGATGAGCATGGTG |
| *MsGRAS120* | AGGAGCTCAATTCCATCGCC | AGGAGTTCGACGGAGACCTT |
| *MsGRAS121* | GGAGGACATCCGTGCTAAGG | ACCAAAACGCTCTCGTCCAT |
| *MsACTIN* | CCCGTTTGGTTCCACATCAA | CCGAACCTGCTGACTTTGTG |
